# Unilateral bi-portal endoscopy for the treatment of thalassemia with extramedullary hematopoietic compression of the spinal cord: Two case reports

**DOI:** 10.1097/MD.0000000000034136

**Published:** 2023-07-28

**Authors:** Jinhua Lin, Xiaofeng Lai, Rui Zhu, Hao Wu

**Affiliations:** a Department of Orthopaedics, First Affiliated Hospital of Jinan University, Tianhe District, Guangzhou City, Guangdong Province, China.

**Keywords:** bi-portal spinal endoscopy, case report, extramedullary hematopoiesis, spinal stenosis, thalassemia

## Abstract

Thalassemia combined with extramedullary hematopoietic spinal cord compression is extremely rare; its ideal treatment is still controversial. Herein, we present 2 cases of thalassemia combined with extramedullary hematopoietic compression of the spinal cord wherein satisfactory results were obtained using unilateral bi-portal endoscopy (UBE).

**Patient concerns:**

Case 1 was of a 43-year-old male who presented with a chief complaint of numbness of the left lower limb since 1-month. Case 2 involved a 23-year-old male who was admitted to the hospital with a chief complaint of numbness in both toes since 3 months and walking instability since 2 weeks. Both cases had a history of being diagnosed with thalassemia.

**Diagnoses:**

Computed tomography and magnetic resonance imaging showed spinal canal space-occupying lesions causing dural compression and spinal stenosis. Postoperative pathology confirmed the spinal canal lesions to be extramedullary hematopoietic tissue.

**Interventions:**

For spinal canal decompression, UBE supplemented by blood transfusion was performed for both cases.

**Outcomes:**

All preoperative symptoms were relieved postoperatively; no recurrence was noted at the 6-month follow-up.

**Lessons:**

Thalassemia combined with extramedullary hematopoiesis can lead to acute spinal cord compression. UBE significantly relieves spinal stenosis symptoms; furthermore, UBE combined with blood transfusion for spinal canal extramedullary hematopoiesis gives satisfactory results, is safe, and has a low risk of spinal cord injury.

## 1. Introduction

Thalassemia is a type of hemolytic anemia caused by gene mutations or deletions in bead protein-regulating genes, resulting in structural abnormalities of hemoglobin. Thalassemia can develop secondary to extramedullary hematopoiesis (EMH) as a compensatory mechanism; EMH commonly occurs in the spleen, liver, and lymph nodes; secondary EMH of the spinal canal is rare. However, secondary EMH of the spinal canal leads to spinal cord compression and requires surgical management. Herein, we report 2 cases of thalassemia combined with EMH causing spinal cord compression who were admitted to our hospital; spinal cord decompression was done by unilateral bi-portal endoscopy (UBE) with satisfactory results.

## 2. Case presentation

**Figure 1.**
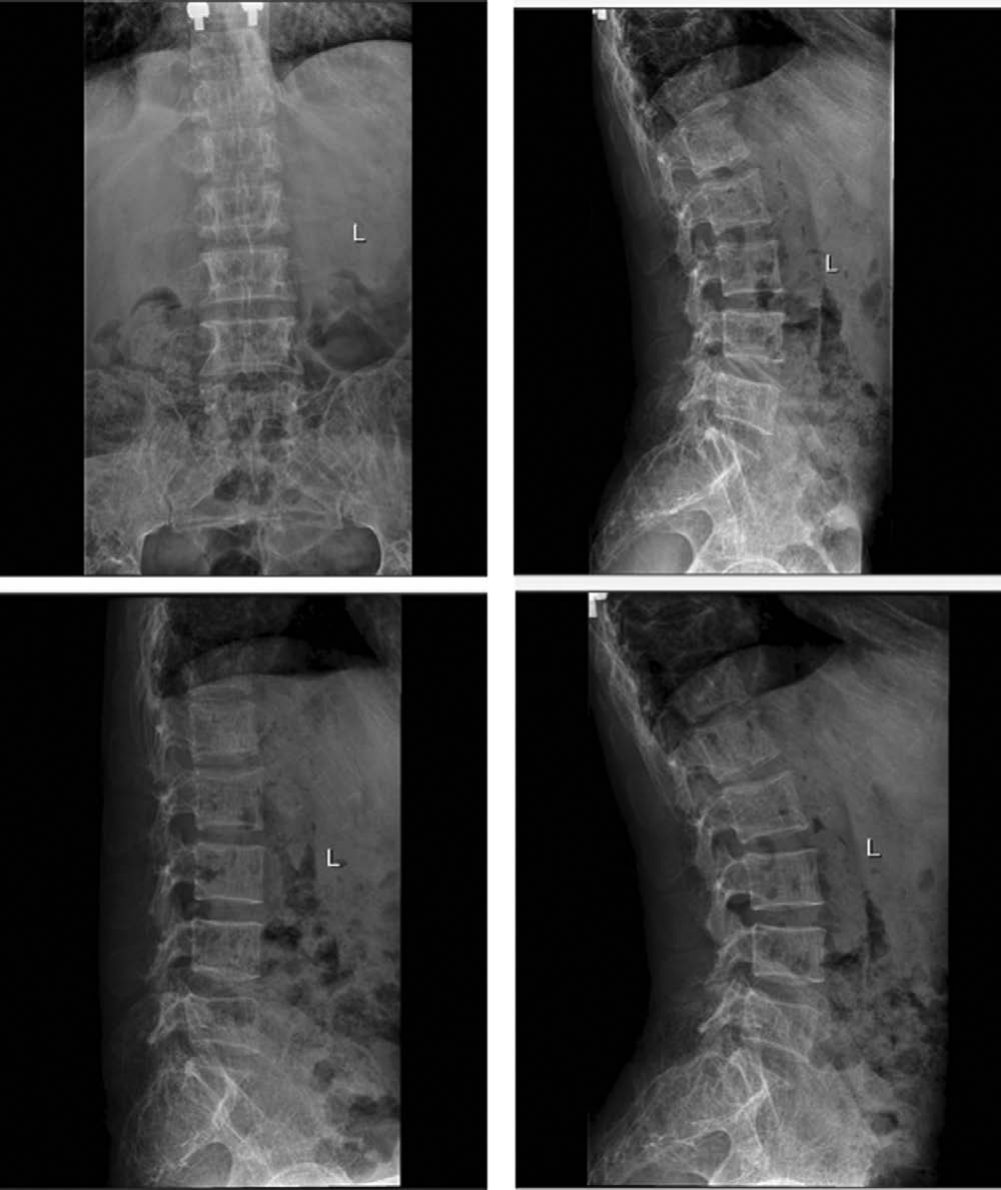
Case 1: No obvious instability of the lumbar spine preoperatively.

**Figure 2.**
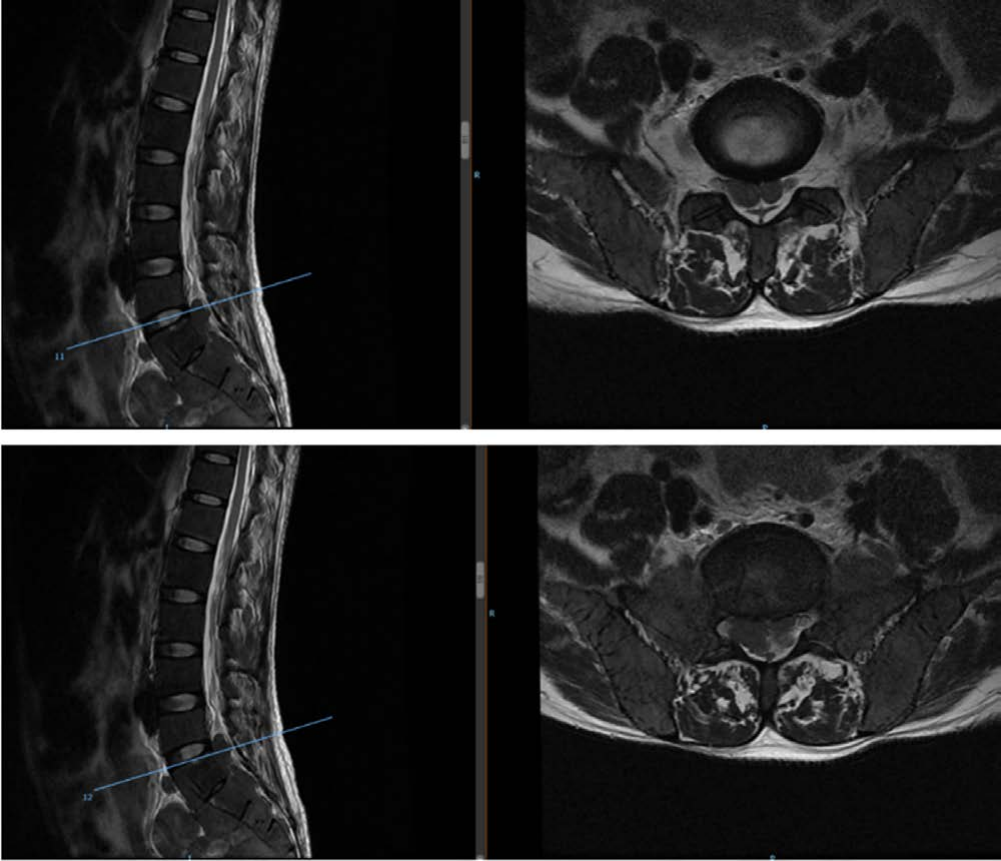
Preoperative magnetic resonance imaging (MRI) T2WI showing L5/S1 and S1/S2 levels secondary to spinal stenosis, and the spinal cord and cauda equina were inflexible.

**Figure 3.**
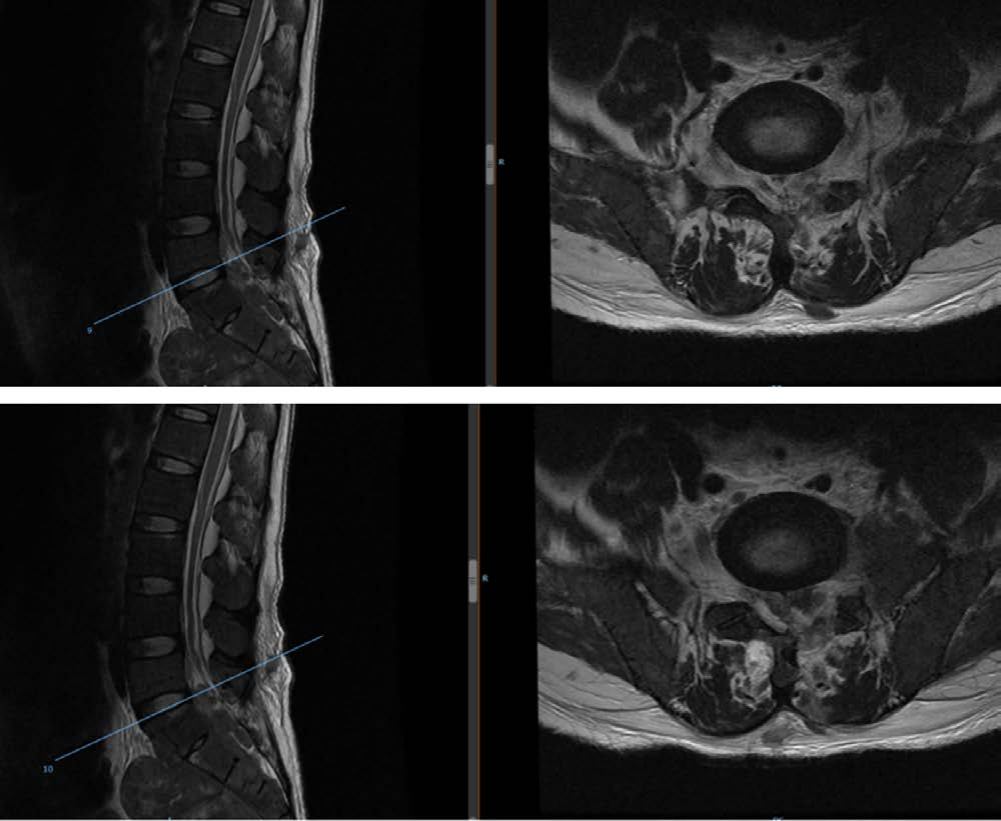
(A) Case 1: Postoperative pathology: Microscopically, bone marrow tissue is seen, significant hyperplasia is noted, the 3 hematopoietic cell lineages are visible, and erythroid hyperplasia is more obvious. (B) Case 2: Postoperative pathology: microscopically, bone marrow tissue with active hyperplasia is observed, and the 3 hematopoietic cell lineages are visible, and the red cell line is more active, which is accompanied by foamy tissue cell reaction.

**Figure 4.**
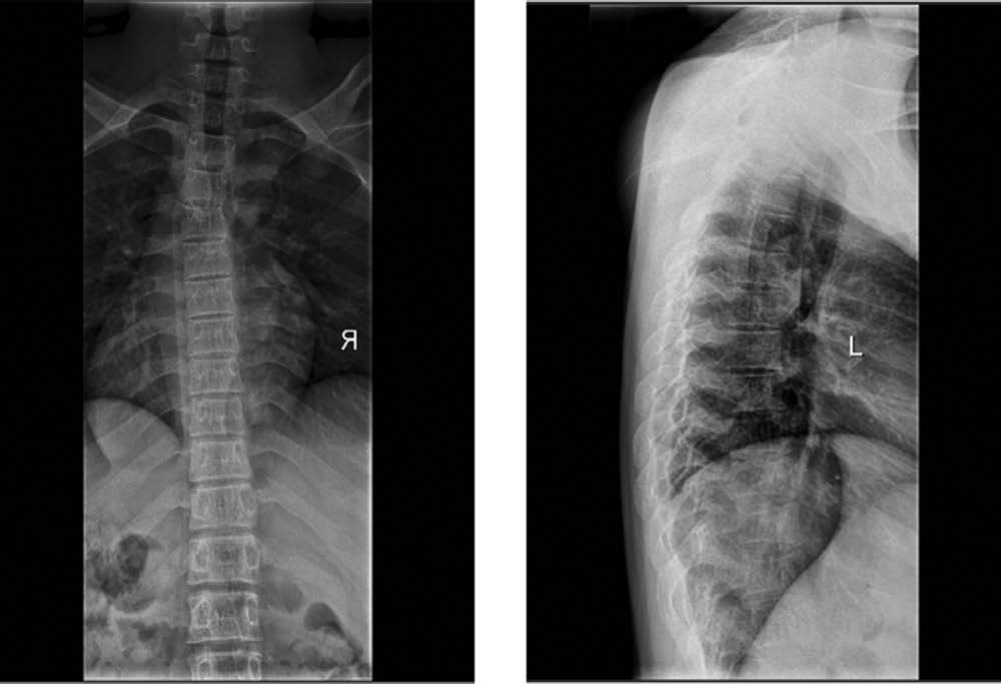
Magnetic resonance imaging (MRI) T2WI postoperatively showing relieved spinal canal compression of L5/S1 and S1/S2.

Case 1: a 43-year-old male from Huizhou City, Guangdong Province, was admitted to our hospital with a complaint of left lower limb weakness with numbness since 1-month. Physical examination revealed numbness in the left plantar area, grade 4 left lower limb muscle strength; a history of thalassemia diagnosis, and thoracic T7-T10 internal fixation. Preoperative magnetic resonance imaging (MRI) showed anemia-related changes in the lumbosacral spine and bilateral iliac bones with multiple intravertebral canal and paravertebral lesions (Figs. 1 and 2). Hence, a diagnosis of EMH in thalassemia with secondary spinal stenosis at several levels and inflexible spinal cord and cauda equina was made. The admission diagnoses were: lumbar spinal stenosis (L5/S1, S1/S2); thalassemia; and postoperative thoracic internal fixation. Subsequently, UBE spinal canal decompression of L5/S1 and S1/S2; extramedullary mass excision; and nerve root exploration and L5, S1, and S2 release were performed under general anesthesia on March 15, 2022; intraoperatively, 2U of red blood cell suspension was infused. Postoperatively, all symptoms were relieved. Postoperative pathological findings included S1/S2, L5/S1 intravertebral canal lesions microscopically, marked proliferation of the bone marrow tissue, and all 3 hematopoietic cell lineages were noted, with pronounced proliferation of the red lineage. Figure 3A presented immunohistochemically the specimen was CD34 (−), CD117 (single, +), CD 3 (small amount, +), CD 20 (small amount, +), MPO (partial, +), TdT (single, +), and approximately 80% Ki-67 (+). Thus, the tissue was extramedullary hematopoietic tissue. Postoperative computed tomography (CT) and MRI showed L5/S1 and S1/S2 spinal canal decompression and resolved extramedullary masses (Fig. 4).

**Figure 5.**
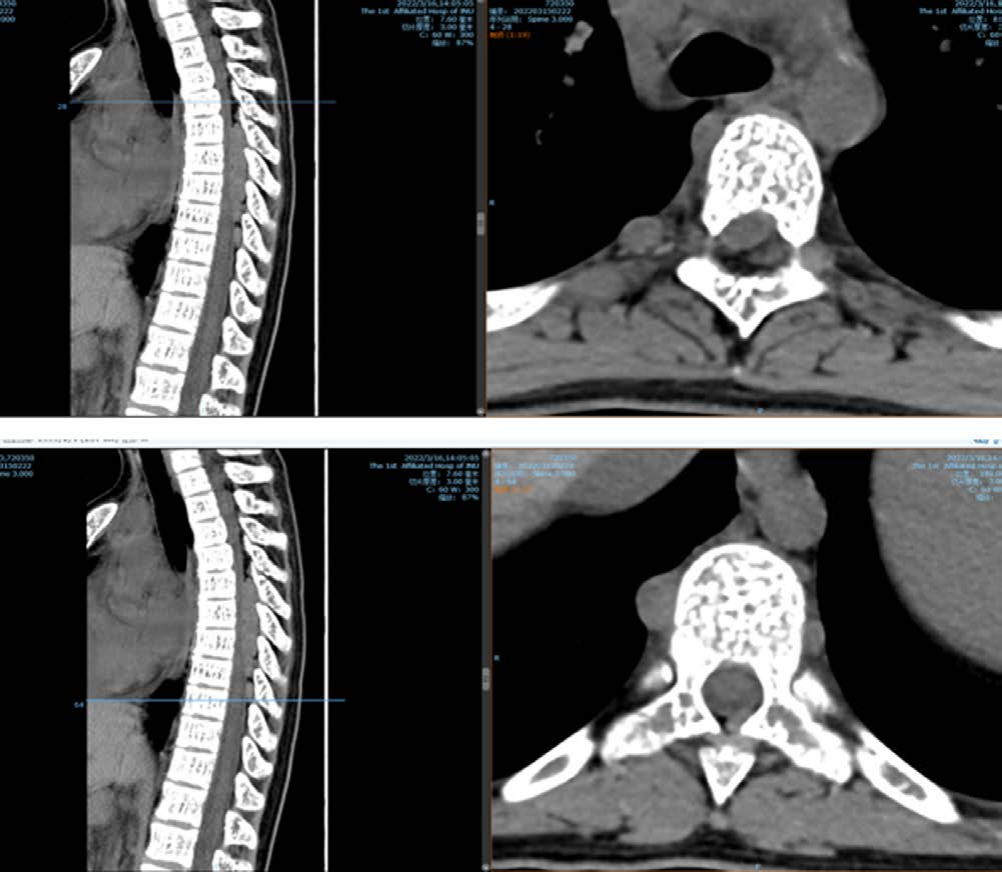
Case 2: preoperative thoracic spine X-ray.

**Figure 6.**
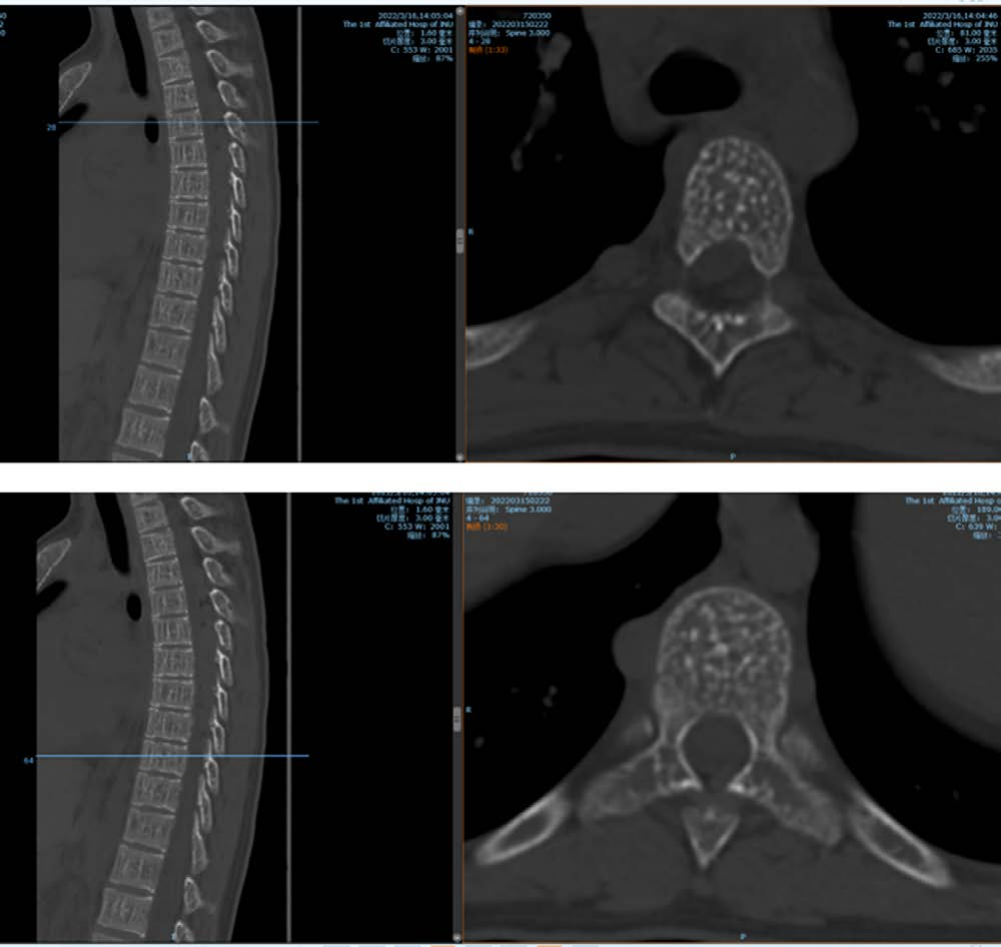
Computed tomography (CT) soft tissue window.

**Figure 7.**
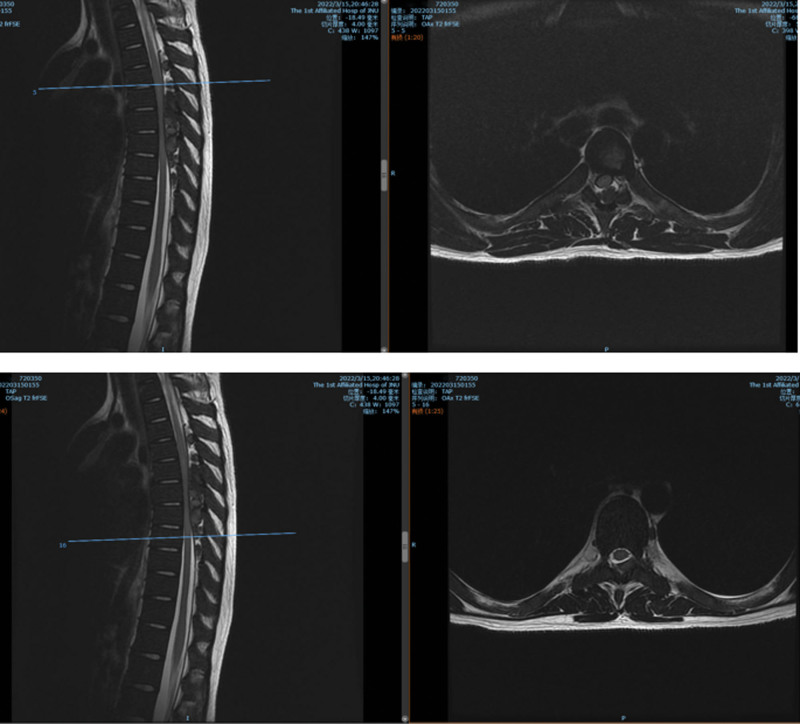
Computed tomography (CT) bone window.

**Figure 8.**
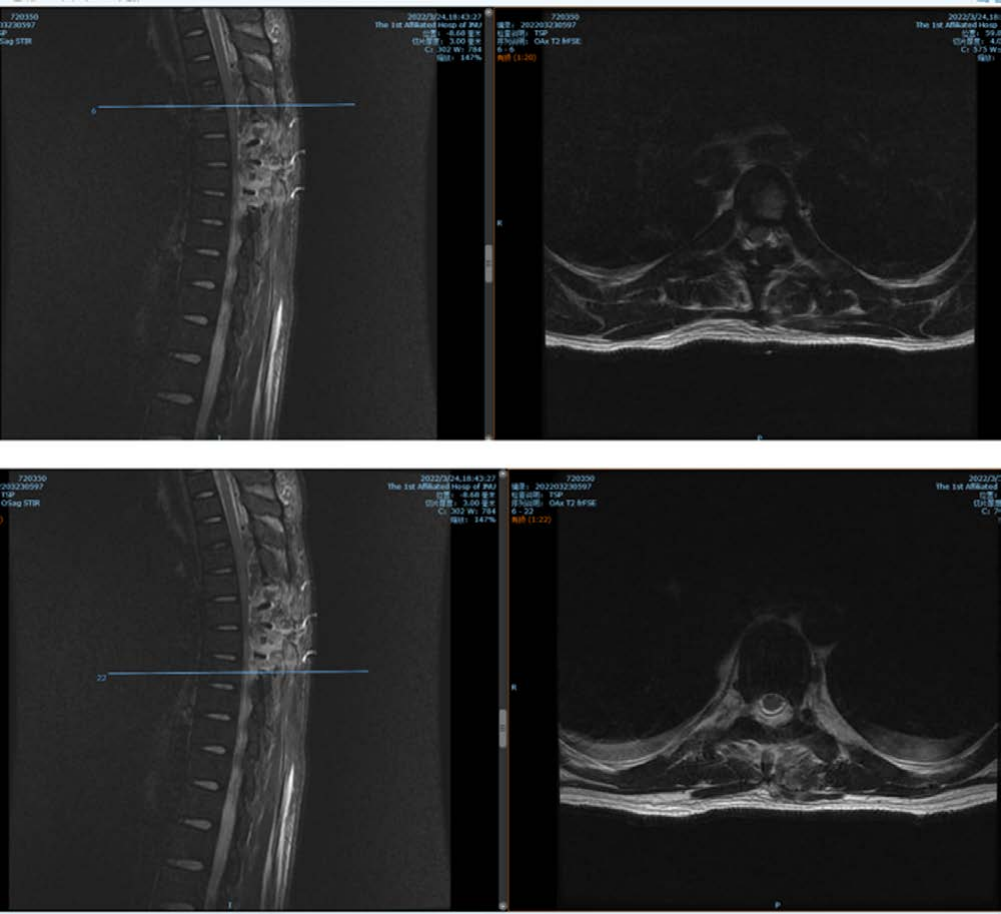
(MRI T2WI) Preoperative computed tomography (CT) and MR showing thoracic spine, rib bone abnormalities, spinal epidural space at the T4-T9 level, left T8 vertebral body, bilateral, multiple space-occupying lesions in the T9 vertebral body. MRI = magnetic resonance imaging.

**Figure 9.**
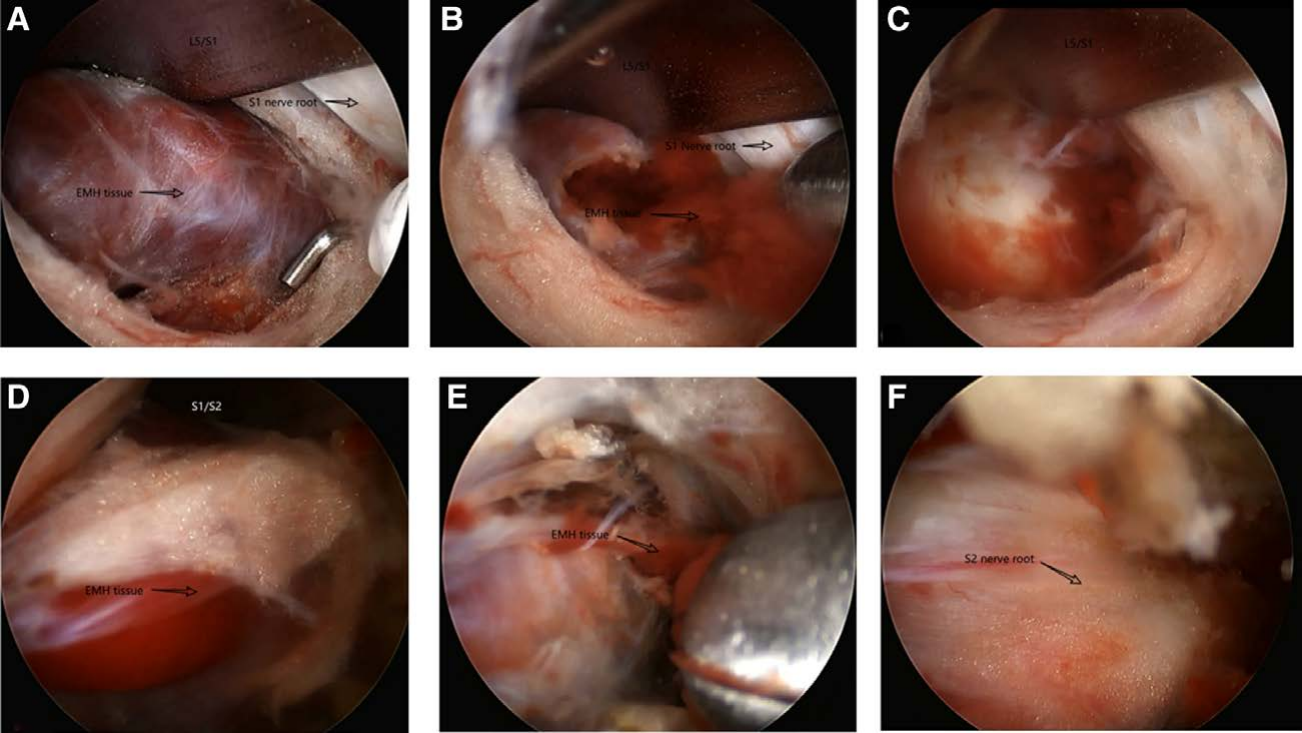
Postoperative magnetic resonance imaging (MRI): T4–8 spinal epidural space-occupying partial resection after the change, soft tissue edema.

Case 2: A 23-year-old male from Foshan City, Guangdong Province, was admitted with a complaint of numbness in both toes since 3 months and walking instability since 2 weeks. Physical examination showed numbness of both toes and grade 4 lower limb muscle strength. The patient reported a history of thalassemia diagnosis. Preoperative thoracic X-ray showed no significant instability (Fig. 5). Imaging revealed thoracic vertebrae and rib bone abnormalities, with multiple masses at the T4-T9 intradural and epidural space, in the distal of the T8 vertebral body, and the T9 vertebral body (Figs. 6,7, and 8); thus, a diagnosis of EMH was made. The admission diagnoses were: thoracic spinal stenosis and thalassemia. UBE was performed under general anesthesia for T4-T8 intraspinal space-occupying resection, semi-laminectomy, and spinal canal decompression on March 22, 2022. Intraoperatively, 2U red blood cell suspension was infused; all symptoms were relieved. The postoperative pathological findings included thoracic spinal canal edema microscopically, actively proliferating bone marrow tissue, and all 3 hematopoietic cell lineages were noted with active red cell lineage and foamy histiocytic reaction. Immunohistochemically the specimen was CD68 (+), CD163 (+), CD1a (−), and S-100 (−) (Fig. 3B). Thus, a diagnosis of EMH was made. Postoperative CT and MRI showed changes after partial resection of an epidural mass in the T4-8 spinal canal and soft tissue edema in the operative area (Fig. 9).

### 2.1. Operation process

**Figure 10.**
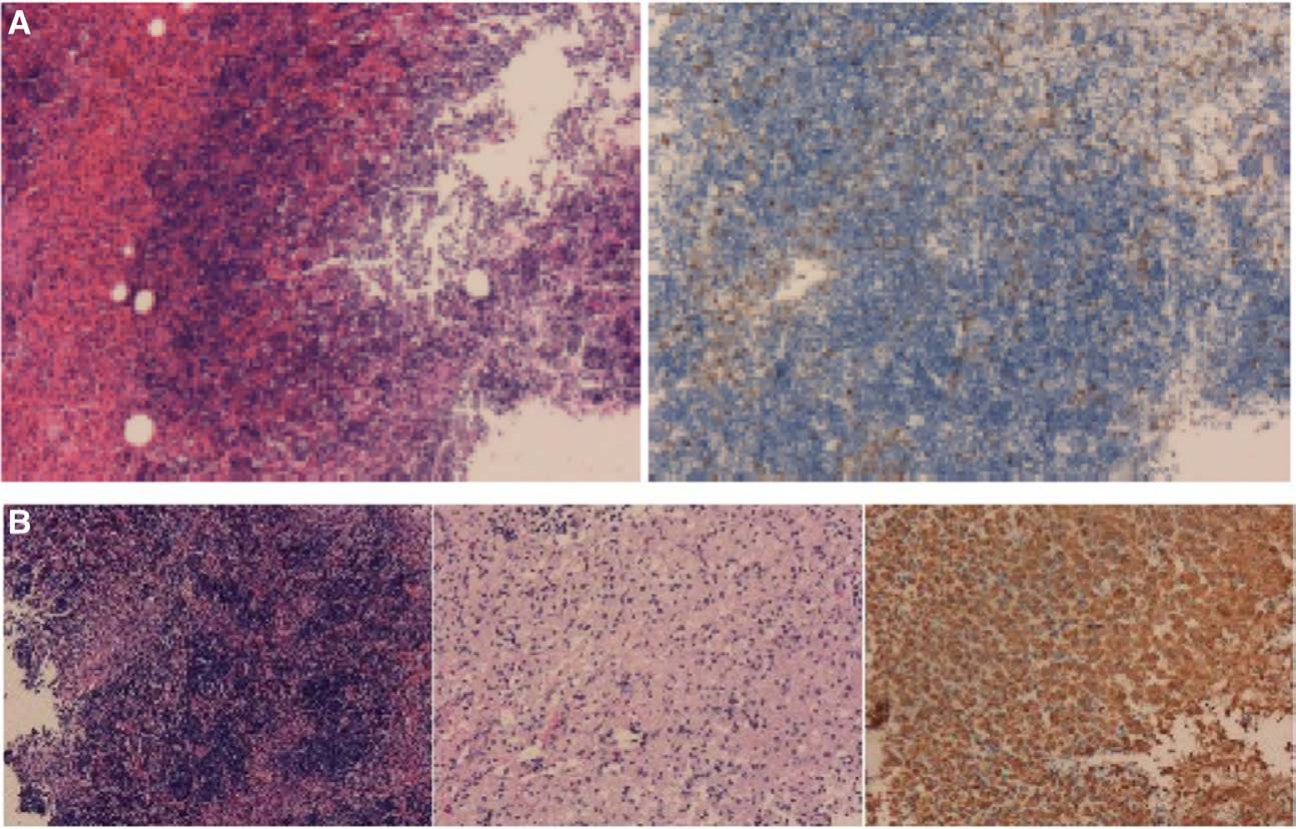
(A–C) Case 1: the L5/ S1 level under UBE microscope, (A) EMH tissue below the S1 nerve root, (B) endoscopic removal of EMH tissue using nucleus pulposus forceps, (C) removal of maximum EMH tissue below the S1 nerve root; in (D and E) Case 1, the S1/S2 level under UBE microscope, (D) spinal canal EMH compressing the S2 nerve root, (E) endoscopic use of nucleus pulposus forceps to grasp the EMH tissue, (F) S2 nerve root compression contact. EMH = extramedullary hematopoiesis, UBE = unilateral bi-portal endoscopy.

In case 1, the patient was placed in a prone position, the L5/S1 intervertebral space was determined and marked by C-arm fluoroscopy. The surgical field was disinfected and covered with sterile film. A 0.7 cm incision was made 1.2 cm left of the L5/S1 intervertebral space. The portal incision was separated using dilators; the fluoroscopic portal was located at the L5/S1 level intervertebral space opening at the L5/S1 vertebral lamina. Next, the L5/S1 interlamina was decompressed to expose the thickened ligamentum flavum, which was removed. The dura mater was retracted centrally and protected, and the left S1 nerve root was exposed. The S1 nerve had severe perineural vascular hyperplasia, and a red, cotton-like mass beneath the S1 nerve was repeatedly grasped. The nerve root edema was relieved, and the intervertebral space was fused. The same method was used to locate the S1/S2 nerve; severe spinal canal compression was present. The S1 and S2 nerve root edema was evident. A hemostatic sponge was used for hemostasis; the mass was removed until the spinal canal and nerve compression were relieved (Fig. 10). The posterior endoscopic system was retrieved to close the wound.

## 3. Discussion

Thalassemia is common in the provinces south of the Yangtze River in China, with the highest prevalence in the provinces of Guangdong, Guangxi, Yunnan, and Hainan.^[1]^ Thalassemia produces ineffective red blood cells that stimulate EMH. Spinal canal compression due to EMH is rare; its incidence is higher in men than in women (Male: Female ≥ 4:1).^[2]^ The 2 patients in this study were Cantonese males, consistent with the epidemiological prevalence of thalassemia and secondary EMH. The origin of spinal epidural extramedullary hematopoietic tissue remains controversial. Sohawon et al hypothesized that the vertebral bone marrow hematopoietic tissue squeezes out through weakened trabecular bone and proliferates in the epidural space.^[3]^ Haidar et al suggested that unmanaged thalassemia can cause chronic hemolytic anemia, which can stimulate the proliferation of the residual hematopoietic cells in the spinal epidural space during the embryonic period. EMH mainly occurs in the lower thoracic spine, followed by the lumbar spine.^[4]^

Spinal EMH is frequently misdiagnosed since it is rare and has nonspecific symptoms. Since its description by Gatto et al in 1954, few studies have reported it.^[5]^ Additionally, its clinical manifestations are nonspecific, such as low back pain, lower limb paresthesia or paralysis, and gait instability. Abbarh et al believed that intraspinal EMH can cause irreversible nerve damage, and MRI is the first choice for diagnosis. Based on a complaint of lower limb weakness and MRI findings in a patient with thalassemia, a preliminary diagnosis can be made. A biopsy to confirm the diagnosis is usually not suggested.^[6]^ Due to excessive vascularization of the EMH tissue in the spinal canal, hemorrhage can occur.^[7]^ EMH can present as lobulated or round tissue shadows with a clear boundary, smooth edge, and different sizes on CT and MRI. The EMH tissue on enhanced CT and MRI shows mild to moderate and uniform enhancement, respectively. Imaging manifestations vary based on active and inactive periods. In the active phase, CT shows isointensity and T1WI shows a low signal and T2WI shows a high signal on MRI; in the inactive phase when there is iron deposition, CT shows high density and T1WI shows a low signal and T2WI shows a high signal on MRI; and in the inactive phase where there is steatosis, CT shows low density and T1WI and T2WI show high signal.^[8]^

Currently, there is no consensus on the most appropriate treatment for EMH-associated spinal stenosis; however, blood transfusion or radiotherapy has been proposed. Spinal cord compression was relieved in 2 cases of spinal canal EMH blood transfusion alone. Blood transfusion improves anemia and directly inhibits EMH.^[9]^ Fareed et al reported a case of spinal canal EMH wherein all nerve compression symptoms resolved after blood transfusion and radiotherapy.^[2]^ However, the radiotoxicity of the compressed spinal cord due to radiotherapy needs further investigation. Radiation-induced tissue edema can cause neurological function deterioration. Moreover, to assess post-radiotherapy immunosuppression, frequent peripheral blood cell counts are required, and total cell depletion may worsen the condition. Hydroxyurea is a ribonucleotide reductase inhibitor that stimulates fetal hemoglobin synthesis and cell reduction to reduce globin chain imbalance, thus reducing ineffective erythropoiesis and EMH; however, limited research is present to support its use.^[4]^

Spinal EMH-associated spinal cord compression can damage the ascending and descending spinal cord pathways and destroy neuronal microcirculation, and early surgical treatment is recommended for patients with activity limitations of the lower limbs.^[10]^ However, traditional open laminectomy induced large trauma and more hemorrhage and aggravates the symptoms of anemia postoperatively, which is not conducive to the patient rehabilitation. Recently, UBE is being widely used in China since it induces less trauma, bleeding, and pain.^[11]^ In this study, both patients had long-term chronic anemia; thus, traditional open surgery to relieve spinal canal compression would have increased the patients circulatory load requirements, thereby affecting the prognosis. The harm of perioperative anemia in orthopedic surgery is mainly manifested as increased postoperative infection rate; prolonged hospital stay; increased postoperative mortality; and delayed postoperative functional recovery, affecting the patient quality of life.^[12]^ In Case 1, UBE was used to decompress the L5/S1 and S1/S2 segments and led to 150 mL blood loss; in case 2, UBE was used to decompress the T4-T8 segments and led to 300 mL blood loss, thus the blood circulation requirements of the patients and the surgical risks were reduced.

Additionally, in UBE the operation portal and observation portal do not interfere with each other. Under a stable light source, the operation field is expanded by spinal endoscopy, and the EMH tissues can be clearly exposed; the operation portal can use conventional instruments, such as a grinding drill to remove part of the lamina, lamina bone biting forceps to remove the hyperplastic ligamentum flavum, nucleus pulposus forceps to remove EMH tissue, to achieve good vision and reduce complications.^[13]^ UBE significantly improved the efficiency of soft tissue removal and releasing the nerve root by the independent operation portal, thus shortening the operation time.^[14]^ Case 2 had thoracic spinal stenosis due to EMH. Due to physiological kyphosis of the thoracic spine, the patient presented with a narrow spinal canal, increased operational difficulty, and a high risk of complications.^[15]^ In thoracic spinal stenosis, the soft tissues are compressed on 1 side, and the paravertebral approach has the least interference with the spinal cord. For central compression, the incision is designed to lean out and extensively resect the synovial joint, and the soft tissue compressing the spinal cord is removed under 30° endoscopic surveillance of the spine.^[16]^ In case 2, a left thoracic paravertebral approach was used to decompress the red, flocculated extramedullary hematopoietic tissue under endoscopic surveillance, and complete nerve root release was performed; post this, the patient symptoms were relieved and muscle strength of both lower extremities increased to grade 4 + without complications, such as cerebrospinal fluid leakage, and with minimal damage to the spinal cord. Gupta et al and De et al each reported a case of epidural EMH with improved muscle strength of the lower extremities after spinal decompression; immediate surgical decompression at the time of acute spinal cord compression symptoms can prevent neurological function deterioration.^[17,18]^

In conclusion, based on the neurological symptoms and MRI findings in a patient with thalassemia, a preliminary diagnosis of spinal EMH can be made; the optimal treatment of spinal EMH needs further study. If the patient develops acute spinal cord compression, UBE can relieve the spinal stenosis and significantly alleviate symptoms. UBE provides a clear visual field and a large operation space, has high efficiency, and induces less bleeding. UBE combined with blood transfusion for spinal EMH has satisfactory efficacy, favorable safety, and a low risk of spinal cord injury.

## Author contributions

**Data curation:** Xiaofeng Lai.

**Methodology:** Rui Zhu.

**Supervision:** Hao Wu.

**Writing – original draft:** Jinhua Lin.
